# Metastatic proinsulin-secreting tumor: a rare cause of persistent hypoglycemia

**DOI:** 10.1210/jcemcr/luag217

**Published:** 2026-09-09

**Authors:** Robert J Heins, John Yazji, Philip Eber, Stefanie Furlan

**Affiliations:** Kansas City University College of Osteopathic Medicine, Kansas City, MO 64106, USA; Kansas City University College of Osteopathic Medicine, Kansas City, MO 64106, USA; Department of Internal Medicine, JFK Medical Center: HCA Florida JFK Hospital, Atlantis, FL 33462, USA; Department of Internal Medicine, JFK Medical Center: HCA Florida JFK Hospital, Atlantis, FL 33462, USA

**Keywords:** proinsulinoma, proinsulin, neuroendocrine, hypoglycemia

## Abstract

Proinsulin-secreting neuroendocrine tumors (NETs) are a rare cause of severe hypoglycemia, characterized by normal or low insulin levels and disproportionate elevations of proinsulin. Clinical features and optimal management of unresectable cases remain poorly defined, in part due to such few cases described in the literature. We describe a 47-year-old female with metastatic large-cell neuroendocrine carcinoma who presented with obstructive jaundice and was incidentally found to have profound, asymptomatic fasting hypoglycemia. Initial insulin and C-peptide levels were within reference ranges, but proinsulin concentrations were markedly elevated. The patient developed recurrent, refractory hypoglycemia requiring high-dose corticosteroids, glucagon, diazoxide, octreotide, and ultimately central-line infusion of 70% concentrated dextrose via total parenteral nutrition. Glycemic control could not be achieved despite maximal medical therapy, and the patient experienced rapid clinical deterioration leading to comfort-focused hospice management. This case highlights the diagnostic challenges of proinsulin-driven hypoglycemia and underscores the need for earlier recognition and improved treatment pathways for patients with proinsulin-secreting NETs.

## Introduction

A proinsulinoma is a pancreatic neuroendocrine tumor (NET) that secretes proinsulin, a prohormone precursor to insulin [1]. Under normal physiology, proinsulin is produced by pancreatic beta cells and is enzymatically cleaved by prohormone convertases into insulin and C-peptide prior to secretion [2]. In proinsulinomas, impaired post-translational processing within neoplastic beta cells leads to premature release of incompletely cleaved proinsulin, producing an elevation in proinsulin with normal or low insulin and C-peptide levels [1, 3]. Fewer than 30 cases have been reported in the literature since 1975, and the true incidence remains undefined [4, 5]. These tumors are diagnostically challenging because proinsulin-driven hypoglycemia often occurs with low or inappropriately normal insulin levels [6]. We present a case of severe, recurrent hypoglycemia due to a proinsulin-secreting tumor in which management was complicated by resistance to conventional medical therapies.

## Case presentation

A 47-year-old female with metastatic neuroendocrine carcinoma presented to the emergency department from her oncologist's office for evaluation of new onset jaundice and clay-colored stools. The patient denied chest pain, shortness of breath, abdominal pain, and notably any symptoms suggestive of hypoglycemia including dizziness, lethargy, or tremor. She had been diagnosed approximately 6 months prior to presentation with a stage IV large cell neuroendocrine carcinoma (Ki-67 100%) of suspected pancreatic origin with hepatic metastasis and was receiving chemotherapy until admission. According to the patient and her outside hematology/oncology team, no prior episodes of hypoglycemia to this degree had been recognized.

Upon initial examination, the patient was afebrile with vital signs within normal limits. Examination showed diffuse jaundice, and scleral icterus. The patient had mild abdominal tenderness, but the abdomen appeared soft with no rebound or guarding. Additionally, cardiac and neurologic examinations were unremarkable.

**Figure 1:**
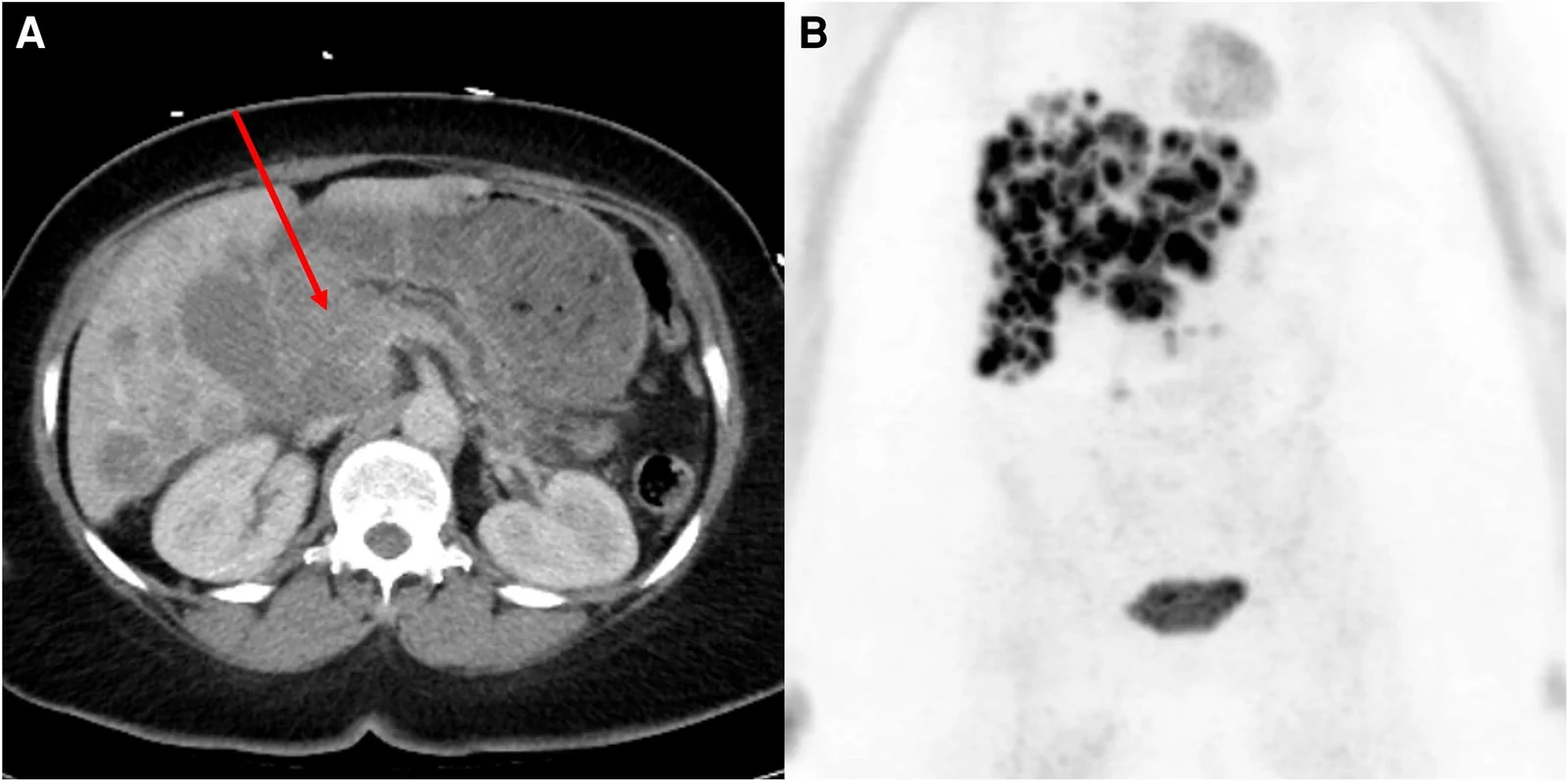
Imaging findings demonstrating metastatic pancreatic malignancy. (A) Axial computed tomography (CT) of the abdomen demonstrating a large hypodense mass in the pancreatic head (red arrow) with associated hepatic metastases (B) Fluorine-18 fluorodeoxyglucose positron emission tomography/computed tomography (PET/CT) showing numerous hypermetabolic lesions throughout the liver consistent with metastatic disease.

Initial laboratory workup revealed severe hyperbilirubinemia of 7.5 mg/dL (SI: 128 μmol/L) (reference range 0.0-1.2 mg/dL [SI: 0-21 μmol/L]), elevated transaminases with an aspartate aminotransferase of 367 U/L (reference range 0-35 U/L) and an alanine aminotransferase of 264 U/L (reference range 0-35 U/L). A capillary plasma glucose obtained on arrival revealed profound hypoglycemia at 25 mg/dL (SI: 1.4 mmol/L) (fasting reference range 70-99 mg/dL [SI: 3.9-5.5 mmol/L]) in the absence of typical symptoms. This finding was subsequently confirmed by multiple venous plasma glucose measurements throughout the hospital course. Computed tomography (CT) of the abdomen without contrast demonstrated progression of the known pancreatic head mass with worsening hepatic metastatic burden (Fig. 1). Subsequent fluorine-18 fluorodeoxyglucose positron emission tomography/computed tomography (PET/CT) revealed extensive hypermetabolic hepatic, pancreatic (6.6 cm head mass), and new pulmonary metastatic disease (Fig. 1).

Throughout the hospital course, the patient experienced recurrent episodes of hypoglycemia of varying severity. Early episodes, including the initial presentation glucose of 25 mg/dL (SI: 1.4 mmol/L), occurred in the complete absence of overt symptoms. As the hospital course continued and episodes became more frequent and severe, the patient began reporting nonspecific malaise and general discomfort coinciding with documented low glucose values, with subjective improvement as glucose levels approached the normal range.

## Diagnostic assessment

During a hypoglycemic episode with a plasma glucose of 34 mg/dL (SI: 1.9 mmol/L), critical sample labs revealed inappropriately non-suppressed insulin and C-peptide and a markedly elevated proinsulin of 974.2 pmol/L (reference range, 0-10 pmol/L) (Table 1). Serum IGF-1, IGF-2, and insulin autoantibody levels were within normal limits, making non-islet cell tumor hypoglycemia and autoimmune hypoglycemia unlikely. Serum acetone was suppressed on serial assessment, consistent with inhibition of lipolysis in the setting of hyperinsulinemic hypoglycemia; beta-hydroxybutyrate, free fatty acids, cortisol, and IGFBP-1 were not measured. Serum lactate was persistently elevated throughout the hospitalization (range 1.9-5.7 mmol/L) without clear resolution prior to the patient's clinical decline.

**Table 1.** Insulin, C-peptide, and proinsulin levels obtained during a hypoglycemic episode.

Laboratory Value	Reference Range	Value Obtained	Expected During Hypoglycemia
**Insulin**	18–150 pmol/L (3–25 μU/mL)	19 pmol/L (3.1 μU/mL)	<18 pmol/L (<3 μU/mL)
**C-peptide**	0.16–1.67 nmol/L (0.48–5.05 ng/mL)	0.49 nmol/L (1.49 ng/mL)	<0.20 nmol/L (<0.6 ng/mL)
**Proinsulin**	0–10 pmol/L (0–88.2 pg/mL)	**974.2 pmol/L (8590.7 pg/mL)**	<5 pmol/L (<44.1 pg/mL)

Expected suppressed values during documented hypoglycemia per the Endocrine Society Clinical Practice Guideline [7].

## Treatment

**Figure 2:**
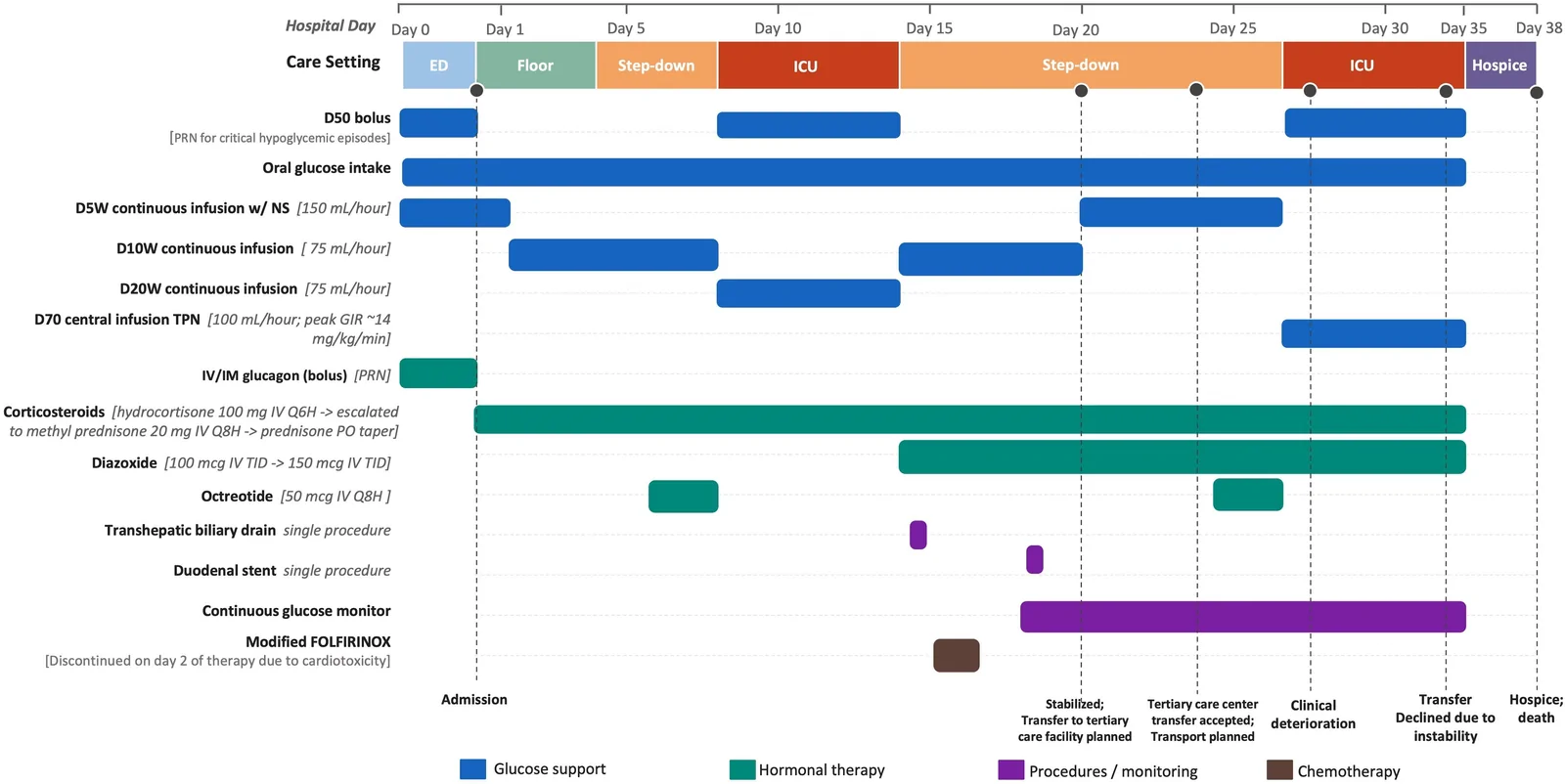
Hospital course and treatment timeline. The patient's 38-day inpatient course is shown across care settings, with overlapping interventions grouped by category. Bracketed values denote doses. Vertical markers indicate key clinical transitions. GIR, glucose infusion rate; TPN, total parenteral nutrition; PO, by mouth; IV, intravenous; IM, intramuscular; PRN, as needed; TID, 3 times daily; Q8H, every 8 hours.

The patient was initially managed with frequent 50% dextrose boluses, intravenous glucagon boluses, and encouraged oral glucose intake (Fig. 2). This resulted in minimal sustained rises in plasma glucose, necessitating transition to a continuous 5% dextrose infusion, subsequently escalated to 10% dextrose after persistent hypoglycemia. Corticosteroids were added given continued hypoglycemia on dextrose infusion alone. The patient was transferred to the intensive care unit for escalation to a 20% dextrose infusion, with concomitant initiation of diazoxide and octreotide and further uptitration of corticosteroids. Given the large amount of fluids this patient had been receiving, urea packets were added to partially offset potential hyponatremia. Despite maximal medical therapy, refractory hypoglycemia persisted throughout the patients’ hospitalization, and the decision was made to initiate a continuous central 70% dextrose infusion at 100 mL/hour, corresponding to a peak glucose infusion rate of approximately 14 mg/kg/min. Subsequently, the patient was transferred to the step-down unit with a newly placed continuous glucose monitor and achieved relative glycemic stabilization with glucose levels consistently above 70 mg/dL (3.9 mmol/L) while transfer to a tertiary care facility was planned.

Additionally, the patient underwent placement of a transhepatic biliary drain for malignant biliary obstruction, resulting in improvement of cholestasis and jaundice. A duodenal stent was subsequently placed to relieve luminal obstruction, which improved oral intake and contributed to glycemic stabilization. Inpatient second-line chemotherapy with modified FOLFIRINOX (oxaliplatin, irinotecan, leucovorin, and 5-fluorouracil) was initiated as part of tumor-directed therapy but was discontinued after 2 days due to cardiotoxicity.

## Outcome and follow-up

Despite escalation of parenteral glucose support, the patient experienced progressive clinical deterioration with recurrent episodes of severe, refractory hypoglycemia, with serum glucose levels repeatedly falling below 10 mg/dL (SI: 0.6 mmol/L). Given the patient's rapid decline, transfer to a tertiary care facility was declined by the outside institution. After goals of care discussion, the patient elected to pursue comfort-focused management and was discharged home with hospice services with patient death occurring the same day.

## Discussion

We report a rare case of a proinsulin secreting NET that was refractory to multiple hypoglycemia treatments. This case represents one of the fewer than 30 documented proinsulinoma cases in the literature and is notable for the requirement of continuous 70% dextrose infusion via central venous access after failure of conventional medical therapy [1, 3-6, 8-11].

Markedly elevated proinsulin levels were identified as the primary driver of this patient's persistent hypoglycemia. Proinsulin retains the ability to activate insulin receptors at approximately 5 to 6% of insulin's potency, with predominant action at hepatocyte receptors that suppresses gluconeogenesis through transcriptional inhibition of phosphoenolpyruvate carboxykinase and glucose-6-phosphatase catalytic subunit [12-15]. Weaker peripheral effects increase muscle glucose uptake and suppress glycogenolysis, lipolysis, and ketogenesis, together producing the characteristic hypoglycemic, hypoketotic, hypofattyacidemic state [13].

The diagnosis was established based on the patient's clinical presentation, markedly elevated serum proinsulin levels, and prior diagnosis of a pancreatic NET. The original biopsy specimen and detailed immunohistochemical staining profile beyond Ki-67 of 100% were not transferable from the outside institution, and retrospective staining was therefore not feasible. Despite this, biochemical and clinical profile functionally defines this as a proinsulinoma regardless of the precise tissue site of origin. Across published cases, proinsulinoma typically presents with hypoglycemia in the setting of normal or low insulin levels and without the obesity or hyperphagia more characteristic of classic insulinomas [4, 5]. Most reported tumors are small, well-differentiated lesions amenable to resection, contrasting sharply with the high-grade, Ki-67 100%, widely metastatic tumor observed here. Reported proinsulin concentrations in prior cases have generally ranged from modest elevations to values in the low hundreds of pmol/L, making the peak level of 974.2 pmol/L observed in our patient among the highest documented to date to our knowledge [3]. Hypoglycemia emerged as a new manifestation despite a known pancreatic neoplasm for more than 6 months, coinciding with documented disease progression and newly identified hepatic and pulmonary metastases. We propose that this carcinoma underwent malignant progression toward a proinsulin-secreting phenotype, a phenomenon previously described by Yoshioka et al, with the extensive hepatic metastatic burden likely contributing further to impaired proinsulin clearance [10]. Progressive deterioration in synthetic hepatic function occurred during the later stages of the hospital course, as evidenced by rising transaminases and bilirubin along with the prolongation of the international normalized ratio. This likely compounded this picture by further reducing endogenous gluconeogenic capacity and impairing hepatic clearance of circulating proinsulin [16].

Definitive treatment of proinsulinomas is surgical resection, which was not feasible in this case given the extent of metastatic disease [5]. Nonsurgical management strategies are poorly characterized and largely adapted from insulinoma management, typically involving dietary modification, dextrose infusions, glucagon, corticosteroids, diazoxide, and somatostatin analogs [17]. Octreotide was similarly ineffective, which is consistent with the known downregulation of somatostatin receptor expression in poorly differentiated neuroendocrine carcinomas with high Ki-67 indices, limiting the pharmacologic target available for somatostatin analog therapy [18]. Paradoxical worsening of hypoglycemia with octreotide has also been previously described in proinsulinoma, further supporting caution with its use [19].

Additional therapeutic strategies described for refractory tumoral hyperinsulinism warrant consideration in future cases. Continuous glucagon infusion has been used as a bridge therapy in selected patients but was not pursued in our patient given the absence of glycemic response to bolus glucagon and the concurrent failure of corticosteroid-driven gluconeogenic stimulation [20]. Alpelisib, a PI3Kα inhibitor that has been reported to attenuate insulin receptor signaling in tumor-induced hypoglycemia, was not available at our institution and was therefore not considered but represents a rational future target in refractory proinsulinoma where standard medical management fails [21].

A focused review of published proinsulinoma cases did not identify comparable use of dextrose therapy in this clinical context, although it is possible that similar approaches have been employed with being formally reported. The absence of established guidelines for maximal glucose infusion rates in refractory proinsulinoma-related hypoglycemia highlights a critical gap in the literature and underscores the need for prospective investigation into novel therapeutic strategies targeting the proinsulin-mediated insulin receptor signaling pathway.

## Learning points

- Proinsulin-secreting NETs are a rare cause of hypoglycemia and may present with normal or low insulin levels despite markedly elevated proinsulin concentrations.
- Measurement of serum proinsulin should be considered in patients with unexplained hypoglycemia when insulin and C-peptide levels are not elevated.
- Proinsulin-driven hypoglycemia results primarily from suppression of hepatic gluconeogenesis due to proinsulin-mediated insulin receptor activation.
- Medical management of unresectable or metastatic proinsulinomas is not standardized and may require multimodal therapy including dextrose infusions, glucagon, corticosteroids, diazoxide, somatostatin analogs, and unusually high concentrations of intravenous glucose to maintain euglycemia.

## Data Availability

Data sharing is not applicable to this article as no datasets were generated or analyzed during the current study.

## Abbreviations

CT: computed tomography
NET: neuroendocrine tumors
PET: positron emission tomography
